# Medication Usage Record-Based Predictive Modeling of Neurodevelopmental Abnormality in Infants under One Year: A Prospective Birth Cohort Study

**DOI:** 10.3390/healthcare12070713

**Published:** 2024-03-24

**Authors:** Tianyi Zhou, Yaojia Shen, Jinlang Lyu, Li Yang, Hai-Jun Wang, Shenda Hong, Yuelong Ji

**Affiliations:** 1 Department of Maternal and Child Health, School of Public Health, Peking University, Beijing 100191, China; 1710306213@pku.edu.cn (T.Z.); 2211210082@bjmu.edu.cn (Y.S.); jinlanglyu@bjmu.edu.cn (J.L.); whjun@pku.edu.cn (H.-J.W.); 2 Peking University Health Science Center-Weifang Joint Research Center for Maternal and Child Health, Beijing 100191, China; 3 Tongzhou Maternal and Child Health Care Hospital of Beijing, Beijing 101101, China; beijingyang2007@126.com; 4 National Institute of Health Data Science, Peking University, Beijing 100191, China; hongshenda@pku.edu.cn

**Keywords:** machine learning, ages and stages questionnaire-3 (ASQ-3), neurodevelopmental abnormality, synthetic minority over-sampling technique (SMOTE)

## Abstract

Early identification of children with neurodevelopmental abnormality is a major challenge, which is crucial for improving symptoms and preventing further decline in children with neurodevelopmental abnormality. This study focuses on developing a predictive model with maternal sociodemographic, behavioral, and medication-usage information during pregnancy to identify infants with abnormal neurodevelopment before the age of one. In addition, an interpretable machine-learning approach was utilized to assess the importance of the variables in the model. In this study, artificial neural network models were developed for the neurodevelopment of five areas of infants during the first year of life and achieved good predictive efficacy in the areas of fine motor and problem solving, with median AUC = 0.670 (IQR: 0.594, 0.764) and median AUC = 0.643 (IQR: 0.550, 0.731), respectively. The final model for neurodevelopmental abnormalities in any energy region of one-year-old children also achieved good prediction performance. The sensitivity is 0.700 (IQR: 0.597, 0.797), the AUC is 0.821 (IQR: 0.716, 0.833), the accuracy is 0.721 (IQR: 0.696, 0.739), and the specificity is 0.742 (IQR: 0.680, 0.748). In addition, interpretable machine-learning methods suggest that maternal exposure to drugs such as acetaminophen, ferrous succinate, and midazolam during pregnancy affects the development of specific areas of the offspring during the first year of life. This study established predictive models of neurodevelopmental abnormality in infants under one year and underscored the prediction value of medication exposure during pregnancy for the neurodevelopmental outcomes of the offspring.

## 1. Introduction

Neurodevelopmental disorders (NDDs) are a diverse group of disorders clinically characterized by deficits in neurodevelopment that manifest in the early childhood [1], persist throughout life, and negatively impact children’s quality of life, health, language expression, cognitive abilities, and social skills. The 5th edition of the Diagnostic and Statistical Manual of Mental Disorders (DSM-5), published by the American Psychiatric Association in 2013, defines NDDs as disorders affecting developmental brain functioning during the developmental period [2]. NDDs encompass a range of types including intellectual disability (ID), communication disorders, autism spectrum disorders (ASD), attention deficit and hyperactivity disorder (ADHD), specific learning disabilities, movement disorders, and other neurodevelopmental disorders [3]. The clinical presentations of these disorders vary, but all can profoundly affect a child’s life.

Early identification, assessment, and treatment of children with NDDs are crucial for improving symptoms and preventing further decline in children with NDDs. Prompt intervention in the early stages of neurodevelopment of children with NDDs, especially those with milder forms, is more likely to enable them to live independently and develop normal social skills [4]. However, due to delayed detection, many children do not receive timely intervention. Currently, severe cases can often be diagnosed at 2 or 3 years of age [5,6], milder cases are typically not diagnosed until 6 or 7 years of age [7], and some cases may never be diagnosed. In Hong Kong, approximately 10% of children with ASD are not identified by the end of first grade [8], and the regular social demands of school life further deteriorate the neurodevelopment of children with NDDs symptoms [9]. Since the pathogenesis of NDDs is not yet clear, early and effective intervention against its controllable risk factors has become the main means of prevention and control of NDDs. Current research evidence suggests that NDDs are disorders caused by pregnancy stress, pregnancy infection or fever, and maternal metabolic syndrome, all of which affect fetal brain development through oxidative stress [10]. Although there is phenotypic heterogeneity in NDDs, many of the influencing factors of NDDs found in previous studies are common to multiple NDDs, such as maternal obesity [11,12], diabetes [13,14], gestational hypertension [15], preeclampsia [15,16], pregnancy stress [17,18], use of antidepressants during pregnancy [19,20], use of acetaminophen-containing drugs during pregnancy [21], preterm birth [22,23], low birth weight [22,24], etc. In addition, the incidence of NDDs is much higher in boys than in girls [25]. This suggests that NDDs have a partially identical pathogenesis early in life.

Several models have been created to predict NDDs during early life stage. These models include using behavioral data [26,27,28], neuroimaging data [29,30,31], genetic data [32,33], individual electronic health records [34,35], and metabolites [36,37] for forecasting NDDs in children. These models have exhibited favorable outcomes, such as identifying potential risk factors, eliminating high diagnostic expenses, and enabling early diagnosis. However, these models majorly use early childhood data and did not evaluate the risk factors during pregnancy. Besides that, some of the data are more difficult to collect and may cause secondary victimization of children. These factors limit the generalization and application of the models.

Recognizing the current significance of early detection, diagnosis, and treatment of children with NDDs, as well as the limitations of previous machine learning models in predicting NDDs. The purpose of this study was to develop a predictive model for children’s neurodevelopment after birth based on pregnancy data, which will help to move the time window for early risk screening for NDDs further forward and has more important public health implications. This study is based on the prospective birth cohort “Peking University Birth Cohort in Tongzhou”. Specifically, Synthetic Minority Oversampling Technique (SMOTE) was first used in this study to correct the category imbalance data. Subsequently, chi-square test, logistic regression and LASSO regression combined with random forest screening were used to screen for risk factors related to one-year-old neurodevelopment. Then, by adjusting the number of variables in the model, the optimal models of children’s neurodevelopment in the five areas were explored, respectively. Finally, the explainable machine learning method was used to explain the model to explore the pregnancy risk factors of children’s early life neurodevelopment.

## 2. Methods

### 2.1. Study Design and Setting

**Figure 1:**
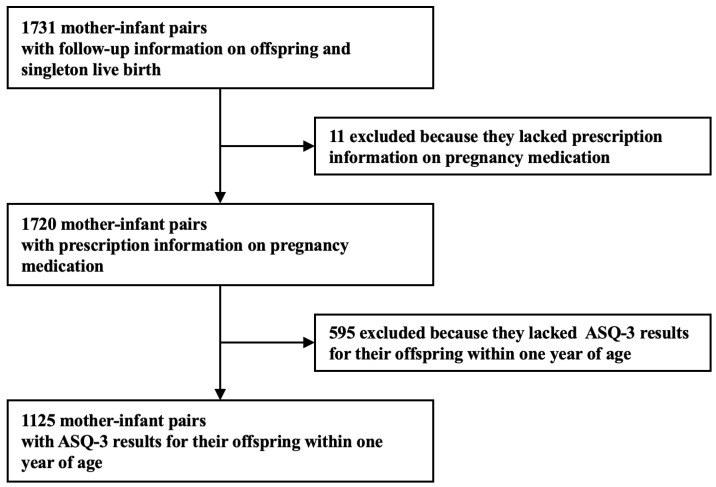
Flowchart of the sample included in the analyses.

This study used a subset data of the Peking University Birth Cohort in Tongzhou (PKUBC-T, https://clinicaltrials.gov/, NCT03814395, accessed on 1 January 2024). PKUBC-T prospectively examined the short- and long-term health effects of maternal exposure during pregnancy on both mothers and their offspring. The detailed description for PKUBC-T can be found in our previous published article [38]. The study initially included 1731 mother–infant pairs with singleton live births, enrolled at the Tongzhou Maternal and Child Health Care Hospital of Beijing, China, during pregnancy and continued in the follow-up study after birth (Figure 1). Mother–infant pairs meeting the following criteria were excluded from participation in this research: lack of data regarding maternal medication usage record during pregnancy and absence of ASQ-3 questionnaire results of the offspring within the first year. Ultimately, a total of 1125 mother–infant pairs were included in this study. The study protocol was approved by the Biomedical Ethics Committee of Peking University (Grant No. IRB00001052-21023). Written consent was obtained from all participants. All the databases for research do not contain personal identifiers and are accessible only by authored investigators.

### 2.2. Definition of Diagnosis Groups

The Chinese version of the Ages and Stages Questionnaire-3 (ASQ-3) was utilized in this research to examine the developmental progress of infants in their first year. ASQ-3 is a reliable and widely used standardized screening tool completed by parents to effectively evaluate children’s development in the areas of communication, gross motor, fine motor, problem solving, and personal-social. The ASQ-3 demonstrates high validity (r = 0.85), two-week retest reliability (r = 0.75~0.82), and internal consistency (α = 0.51~0.87) [39]. Pregnant women completed the questionnaire under the guidance of medical personnel, assessing the infant’s performance. Children’s developmental outcomes in the five ASQ-3 areas were classified as “normal” or “abnormal” based on their scores and the corresponding cutoff values.

### 2.3. Maternal Medications Exposure during Pregnancy

After completing the child’s one-year follow-up, the exposure of pregnant women to medications during pregnancy was assessed by using both in-patient and out-patient prescription data obtained from Tongzhou Maternal and Child Health Care Hospital of Beijing. All medications prescribed to pregnant women from the start of their last menstrual period until their delivery were abstracted for this study. We processed the prescription data by consolidating different dosage forms of the same medication, such as tablets, oral liquids, and capsules, into a single medication. Medications were also grouped based on their ingredients; for example, aminophenol mephedrone oral solution and acetaminophen tablets, which both contain acetaminophen as the main ingredient, were combined into the “acetaminophen” exposure. Additionally, medications that were used less than 1% of the time in the mother included in the study were dropped. The study ultimately encompassed 105 medications.

### 2.4. Definition of Maternal Characteristics

This paper is based on a prospective cohort study in which a questionnaire was used to collect the information needed for the study during the follow-up of pregnant women in early, mid- and late-pregnancy. Specifically, maternal sociodemographic and behavioral information were collected by uniformly trained healthcare workers at the Tongzhou Maternal and Child Health Care Hospital of Beijing through a questionnaire, including (1) demographic information, such as age, pre-pregnancy body mass index (BMI), number of people in the household, and annual household income; (2) behavioral patterns during pregnancy, such as smoking (Non-Smoker vs. Ex-Smoker vs. Smoker), alcohol consumption (never drink vs. ever drink), folate supplementation (Never supplemented folate vs. Formerly supplemented folate vs. Currently supplemented folate), iron supplementation (Never supplemented iron vs. Formerly supplemented iron vs. Currently supplemented iron), anxiety levels (Never anxiety vs. Subthreshold anxiety vs. Severe anxiety), and sleep quality (Normal vs. Poor); (3) exposure to environmental factors during pregnancy, such as life stress (whether or not they have worked overtime or night shifts), chemical factors exposure (whether or not they have come into contact with pesticides or insecticides, organic solvents, nail polish, hair dyes/perms, lipsticks during work time), physical factors exposure (whether or not they have come into contact with radioactivity, heavy metals, high temperatures, noise during work time), exposure to biological factors (whether or not they have come into close contact with cats and dogs during work time).

According to the Chinese Guidelines for the Prevention and Control of Overweight and Obesity in Adults [40], this study defines pre-pregnancy BMI < 18.5 kg/m^2^ as underweight; 18.5 ≤ BMI < 24 kg/m^2^ as normal weight; and BMI ≥ 24 kg/m^2^ as overweight and obesity. Additionally, according to the definition of the American College of Obstetricians and Gynecologists (ACOG) [41], women older than 35 years of age were defined as “advanced maternal age”. Finally, the Pittsburgh Sleep Quality Index (PSQI) was utilized to assess the sleep quality of mothers and stratified the population based on a cutoff score of 7, where scores greater than 7 were deemed indicative of poor sleep quality.

### 2.5. Statistical Analysis

Prior to data analysis, the data were rigorously preprocessed to ensure the reliability and accuracy of the dataset used in the analysis phase. We interpolated maternal pregnancy missing data using either the multitude or the median, and for missing data in specific areas of the offspring, we interpolated them using the mean of the other area scores for that individual. In addition, we used the Tukey’s fences method to identify outliers in maternal height and weight data and analyze the potential causes of these outliers in order to back-correct and obtain accurate heights and weights, providing a more complete and reliable dataset for subsequent data analysis and model training.

Since the endings in this study were dichotomous variables, we conducted chi-square (Fisher’s exact test), logistic regression, and LASSO regression to identify statistically significant variables associated with the outcomes in five areas within one year age on the three databases generated from the oversampling methods. These significant variables were categorized into “base variables” and “medication variables” based on their property. Furthermore, we utilized the random forest model to rank the importance of the medication variables screened in the previous step and identify the top 10 and top 50 medications in terms of importance, denoted as “Top 10 medications”, ”Top 20 medications”, and “Top 30 medications”. In our analysis, we labeled the model using only the base variables as “model 1”, the model using the base variables plus the top 10 medications as “model 2”, the model using the base variables plus the top 20 medications as “model 3”, the model using the base variables plus the top 30 medications as “model 4”, the model using base variables plus all medication variables as “model 5”.

Predictive modeling of neurodevelopmental abnormality in the five areas of the child within the first year of life was carried out using Support Vector Machine, Random Forest, and Artificial Neural Network models with combinations of the base variable plus all medications. There is a category imbalance in the data of this study, i.e., there is a large difference in the number of normal and problematic children in the total sample. The traditional prediction model is mainly used for classification of data with balanced distribution among categories, and the classification effect decreases dramatically in the category imbalance data, i.e., the traditional prediction model is more sensitive to the data with higher proportion, and the model will be biased to give better results to the categories with higher proportion in the training process. Past studies have demonstrated that machine-learning models based on the SMOTE approach can achieve good predictive results for category imbalance data for neurodevelopmental disorders in children [42]. SMOTE is different from other oversampling algorithms in that SMOTE balances the dataset by synthesizing new minority class samples by incorporating information from surrounding sample points to increase the number of minority class samples [43]. Therefore, in this study, the SMOTE oversampling technique was used to handle case imbalances in five areas. Based on the combined performance, Artificial Neural Networks was chosen as the best predictive method.

We used repeated 10-fold cross-validation for class imbalance data for all model construction. Specifically, we divide the data into a training set and a test set according to the ratio of 9:1, train the model in the training set where SMOTE corrects the class imbalance, and test the model performance in the test set that is not corrected by SMOTE to obtain a stable prediction effect. The models were constructed in each of the five areas using combinations of variables corresponding to models 1-5 combined with the ANN algorithm to explore the optimal combination of variables for each of the five areas. The constructed models were then optimized using grid search. The optimized models were used to calculate the probability of disease in each of the five areas for a child at one year of age, and the calculated probabilities were utilized to create a predictive model for neurodevelopmental abnormalities in any of the child’s areas. Finally, interpretable machine-learning methods (using fastshap package) were used to calculate SHapley Additive exPlanations (SHAP) value to explain the contribution of the variables in the predictive models of five areas. SHAP is a model-independent explainable machine-learning method designed to provide an explanation of the prediction process of machine learning. It is based on Shapley values from game theory and provides insights into the model decision-making process by calculating the contribution of each feature to the model prediction [44]. Due to its highly generalizable and intuitive features, SHAP has become one of the most commonly used interpretable machine-learning methods [45]. R software, version 4.2.1 (R Foundation) was used to perform all analyses [46].

## 3. Result

As shown in Table 1, of 1125 participants (572 (50.8%) male), the final sample included 55 (4.9%) children with communication disorder, 83 (7.4%) with gross motor disorder, 26 (2.3%) with fine motor disorder, 33 (2.9%) with problem solving disorder, and 77 (6.8%) with personal-social disorder. Tables S1–S5 display the predicting performance for the five areas neurodevelopmental abnormality under one year of age, utilizing various models combined with full variables under the SMOTE oversampling technique. A comprehensive comparison reveals that the neural network model established under the SMOTE oversampling method exhibits higher sensitivity, thus the subsequent training of neural network models using the SMOTE-corrected dataset was conducted.

**Figure 2:**
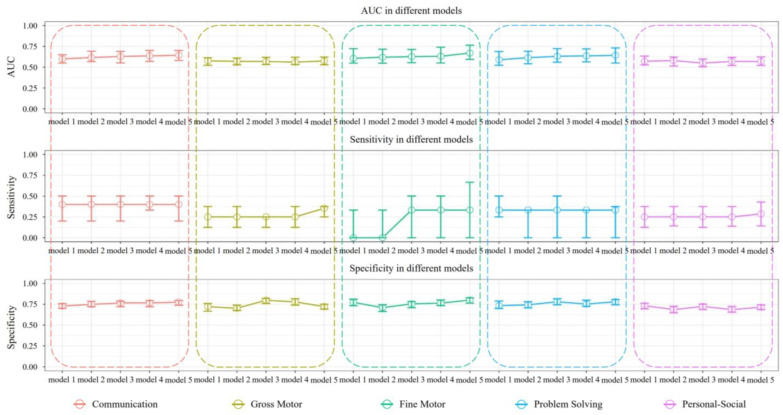
Predictive efficacy of different neural network models in five areas.

The results of repeated 10-fold cross-validation are presented in Table 2. The neural network model was trained with a SMOTE-corrected training set, and the performance of the model was validated on the test set. In the communication area, the predictive model discriminated between communication disorder infants vs. normal infants with an AUC of 0.644 (IQR: 0.581, 0.701). Figure 2 shows the variation in the predictive ability of the model with an increasing number of predictive variables. As the number of predictor variables in the model increases, the model sensitivity is always 0.400, while the specificity tends to increase until model 5 reaches a maximum of 0.776 (IQR: 0.738, 0.794).

In the gross motor area, the predictive model discriminated between gross motor disorder infants vs. normal infants with an AUC of 0.577 (IQR: 0.523, 0.613). Figure 2 demonstrates that as the predictor variables increase in the model, the sensitivity of the model peaks at 0.354 (IQR: 0.250, 0.375) in model 5. The model’s specificity initially increases, then decreases.

In the fine motor area, the predictive model discriminated between fine motor disorder infants vs. normal infants with an AUC of 0.670 (IQR: 0.594, 0.764), which indicates fair discrimination. The sensitivity of the model increased as the predictor variables in the model increased, reaching a maximum of 0.333 at model 3-5. The model’s specificity initially decreases, then increases.

In the problem-solving area, the predictive model discriminated between problem solving disorder infants vs. normal infants with an AUC of 0.643 (IQR: 0.550, 0.731), which indicates fair discrimination. As the number of predictor variables in the model increased, the sensitivity is always 0.333, and there was no trend in specificity with an increasing number of variables, with model 5 performing best overall.

In the personal-social area, the predictive model discriminated between personal-social disorder infants vs. normal infants with an AUC of 0.569 (IQR: 0.521, 0.624). No significant trend in specificity was observed as the number of variables in the models increased. The sensitivity of the model peaks at 0.286 (IQR: 0.143, 0.429) in model 5.

Table 3 shows the prediction performance of the final model. After the grid search optimization of model 5 with five areas, the five optimized models were used to predict the probability of disorder development of five areas when children were one year old, and the final prediction model was established for “abnormal development of any area of children” by using the predicted probabilities. The results showed that the model performed best in the test set when size = 4 and decay = 0.2, the sensitivity of the model for one-year-old children with neurodevelopmental abnormalities was 0.700 (IQR: 0.597, 0.797), the AUC was 0.821 (IQR: 0.716, 0.833), the prediction accuracy was 0.721 (IQR: 0.696, 0.739), and the specificity was 0.742 (IQR: 0.680, 0.748). The accuracy, sensitivity, specificity, and AUC values of the model are all high, which means that the model has demonstrated excellent accuracy and reliability in distinguishing children with neurodevelopmental abnormalities from healthy children.

Table 4 shows the five most important variables for each area, which were obtained by analyzing Model 2 for each of the five areas with interpretable machine learning method. In terms of sociodemographic and behavioral variables during pregnancy, it has been observed that factors such as family income, sum of people, chemical exposure, and maternal pre-pregnancy BMI, all have an impact on the neurodevelopment in various areas of the offspring. Moreover, folate, physical exposure, PSQI, and anxiety have been found to affect the neurodevelopment of specific areas in the offspring. In terms of medication variables, this study has identified a range of medications that affect specific areas in the offspring within the first year of life. For instance, maternal exposure to acetaminophen and chuanbai anti-itch wash during pregnancy was associated with gross motor abnormality of the offspring within the first year of life. Similarly, maternal exposure to ferrous succinate, hydroxyethyl starch sodium chloride, midazolam injection, and ropivacaine mesylate injection during pregnancy was linked to fine motor abnormality of the offspring at one year of age. Furthermore, maternal exposure to blue scutellaria oral liquid and calcium acetate granules during pregnancy was associated with problem solving abnormality and personal-social abnormality, respectively, within the first year of life.

## 4. Discussion

This study aims to develop a predictive model for neurodevelopment in the early years of a child’s life using mothers’ sociodemographics, maternal behavior during pregnancy, and medication prescriptions during pregnancy as predictive variables. We developed and compared four prediction models in each of the five areas using both questionnaire and clinical data from Tongzhou Maternal and Child Health Care Hospital of Beijing, China. Our neural network model performed the best among all models. The AUC in the five regions were 0.644, 0.577, 0.670, 0.643, and 0.580, respectively. The AUC of the final model for any of the area disorders in children at one year of age was 0.821. This research further attempted to explore which variables considered in the predictive model were more influential in predicting the outcomes using an interpretable machine learning approach. It was discovered that among sociodemographic and behavioral variables, family income, sum of people, chemical exposure, and maternal pre-pregnancy BMI, which are pregnancy exposures, can have a greater effect on the neural development of specific areas of the offspring in the first year of life. And folate, physical exposure, PSQI, anxiety, which are factors in which maternal exposure can have an effect on the neurodevelopment of specific areas in the offspring during the first year of life. Among the medications, we found acetaminophen, blue scutellaria oral liquid, calcium, chuanbai anti-itch wash, ferrous succinate, hydroxyethyl starch, midazolam, and ropivacaine mesylate to be effective in the development of the offspring. These medications exposure can affect the development of specific areas in the first year of life of the offspring.

The predictors identified in our predictive models are biologically sound and consistent with the findings of previous studies. Similar to previous studies, the present study identified variables in which family income, sum of people, chemical exposure, pre-pregnancy BMI, folate, physical exposure, PSQI, and anxiety during pregnancy had a greater impact on offspring neurodevelopment. Previous studies have reported that low family income [47,48], small family size [49], chemical exposure during pregnancy [50,51], abnormal pre-pregnancy BMI of the mother [52], no folic acid supplementation during pregnancy [53,54], physical exposure during pregnancy [55], poor sleep quality [56], and anxiety [57] are more likely to have neurodevelopmental abnormality infants. It should be noted that there are limited studies on the quality of maternal sleep during pregnancy and the neurodevelopment of the offspring. Only one study has confirmed that maternal poor sleep quality during pregnancy is linked to ADHD symptoms in the offspring at the age of four [56]. The findings of this study underscore the importance of monitoring and intervention of the above risk factors in pregnancy in actual clinical work. By intervening with these risk factors, it will help reduce the risk of neurodevelopmental abnormalities in early childhood, which in turn can improve long-term health outcomes. In addition, this study provides further evidence on the relationship between maternal sleep quality and early childhood neurodevelopment, suggesting that healthcare professionals should focus on the control of maternal sleep quality in prenatal care. The findings of this study contribute to more comprehensive intervention strategies for the care of children during pregnancy to optimize early childhood development and health.

Among the medication variables, our findings on acetaminophen, calcium acetate, and ferrous succinate align with past research. We observed that exposure to these medications during pregnancy is associated with the development of gross motor, personal-social, and fine motor skills in offspring during the first year of life, respectively. Previous studies have demonstrated that the pharmacological action of acetaminophen is to control the subsequent inflammatory response by specifically inhibiting type II cyclooxygenase (COX-2) [58]. In addition, acetaminophen specifically inhibits prostaglandin synthesis in the brain [58]. However, appropriate levels of prostaglandins in the brain are fundamental to many physiological functions of the brain. Prostaglandin D2 is an important influence on brain development and function [58]. Prostaglandin E2 affects the excitation frequency of neurons in the hippocampus and is involved in anti-inflammatory responses in the brain [58]. Inadequate brain prostaglandin levels also adversely affect learning [59] and cerebellar development [60]. Acetaminophen can be transferred from the mother to the offspring through umbilical cord blood [61]. Additionally, due to infants’ limited metabolic capacity, acetaminophen and its harmful byproducts may persist in their bodies for an extended period [62]. This allows acetaminophen to affect fetal brain development by decreasing prostaglandin synthesis in the fetal brain even at safe adult dose exposures. A meta-study by Wang and colleagues revealed that lower levels of vitamin D increased the risk of ASD by 54% [63], which may be linked to the impact of calcium on early neurodevelopment in offspring [64]. Moreover, ferrous succinate is biologically plausible to affect the development of fine motor skills in offspring through the treatment of iron deficiency anemia (IDA) and preventive iron supplementation, subsequently preventing impaired neurodevelopment [65].

We have discovered, for the first time, a correlation between maternal exposure to blue scutellaria oral liquid, midazolam, and ropivacaine mesylate during pregnancy and the neural development of offspring within their first year of life. Our research delved deeper into the literature on these identified medications. Past studies have found scutellaria, one of the active ingredients in blue scutellaria oral liquid, to be potentially therapeutic for ADHD [66], which has also been demonstrated in animal studies [67]. Midazolam is widely used as a sedative and anesthetic, and animal experiments have shown that exposure to midazolam early in life may lead to alterations in brain structure and function [68,69,70,71], by affecting mitochondrial function, cytoskeletal assembly, and other processes [72,73,74,75]. Ropivacaine mesylate, a surgical anesthetic, has been the subject of few studies analyzing its neurotoxicity, and we found only one study in animals. The study reveals that ropivacaine mesylate is potentially neurotoxic and exerts its effects by upregulating Fas/FasL expression in pheochromocytoma PC12 cells [76].

In addition, we found for the first time that exposure to hydroxyethyl starch sodium chloride and chuanbai anti-itch wash during pregnancy was associated with the development of fine motor and gross motor areas, respectively, in offspring within one year of age. However, past studies have not revealed the neurotoxicity of hydroxyethyl starch [77], and there are no articles examining the association between chuanbai anti-itch wash and neurodevelopment of offspring. This correlation might be due to the indications of these medication, such as intrauterine infections and hemorrhagic shock.

The present study could potentially add to the existing literature in a number of ways. To our knowledge, most previous studies have utilized predictors specific to early childhood (e.g., neuroimaging images, child behavioral data, electronic medical records) to forecast neurodevelopmental outcomes in offspring, and only a few have utilized predictors traced back to the pregnancy period. Acquiring predictors of early childhood is time-consuming and late for early prevention. This study was the first to analyze how sociodemographic and behavioral factors collected during maternal pregnancy, and the use of medications during pregnancy predict neurodevelopmental abnormality in offspring within the first year of life. This study pushes the timeframe for identifying neurodevelopmental abnormality back to the first year of life, holding significant implications for public health.

The investigation solely contained records of hospital in-patient and out-patient prescriptions issued during the mother’s pregnancy and did not encompass data on the actual medication consumed by the mother. Some pregnant women might have ingested less actual medication than prescribed, considering the negative effects of medication taken during pregnancy on the offspring. As a result, we have underestimated the actual medication effects in the right direction, and the real medication effects should be stronger than the results obtained in this study. Second, this study did not include information on the week of gestation when the medication was taken by pregnant women, but some medications have a recommended dosage time for pregnant women. For instance, Ferrous Succinate is recommended for pregnant women after the third month of pregnancy, which to some extent controls for the bias caused by the lack of dosing time. Third, the ASQ-3 questionnaire for children was completed by mothers under professional guidance and in conjunction with the children’s performance in the field; however, the hospital environment may have impacted the children’s performance. To ensure the accuracy of the questionnaire, a room specialized for questionnaires was established to eliminate external interference. Fourth, for infants who are in the process of growth and development, the results of ASQ-3 are only indicative and do not reflect the real development in the later stages of life. Lastly, this study only concentrates on the neurodevelopment of children up to 1 year of age, and the trajectory of children’s neurodevelopment from 0-6 years of age needs to be further explored.

## 5. Conclusions

This study established predictive models of neurodevelopmental abnormality in infants under one year using maternal sociodemographic, behavioral, and medication-usage information during pregnancy as features. This study underscores the prediction value of medication exposure during pregnancy for the neurodevelopmental outcomes of the offspring. More predictive models need to further incorporate information from the patient’s gynecologist to provide more accurate results for child neuropsychological development, including clinical diagnosis, obstetric information, and imaging data.

## Figures and Tables

**Table 1:** Basic Characteristics of the Study Subjects.

Variable	Overall (%)
Total	1125
Male = yes	572 (50.8)
Advanced maternal age = yes	128 (11.4)
Maternal pre-pregnancy BMI	
Low	91 (8.1)
Norm	677 (60.2)
Fat	357 (31.7)
Sum of people (mean (SD))	3.35 (1.31)
Family income (mean (SD))	17.03 (13.89)
Smoke	
Non-Smoker	1058 (94.0)
Ex-Smoker	61 (5.4)
Smoker	6 (0.5)
Alcohol = ever drink	34 (3.0)
Folate	
Never supplemented folate	237 (21.1)
Formerly supplemented folate	90 (8.0)
Currently supplemented folate	798 (70.9)
Iron	
Never supplemented iron	1079 (95.9)
Formerly supplemented iron	8 (0.7)
Currently supplemented iron	38 (3.4)
Anxiety	
Never anxiety	1011 (89.9)
Subthreshold anxiety	110 (9.8)
Severe anxiety	4 (0.4)
PSQI = yes	140 (12.4)
Pressure exposure = yes	109 (9.7)
Chemical exposure = yes	481 (42.8)
Physical exposure = yes	303 (26.9)
Animal exposure = yes	271 (24.1)
Medication types taken (mean (SD))	17.23 (5.96)
ASQ-3 result	
Communication disorder = yes	55 (4.9)
Gross Motor disorder = yes	83 (7.4)
Fine Motor disorder = yes	26 (2.3)
Problem Solving disorder = yes	33 (2.9)
Personal-Social disorder = yes	77 (6.8)

**Table 2:** Predictive efficacy of different neural network models in five areas.

Area	Model	AUC Median (25%, 75%)	Specificity Median (25%, 75%)	Sensitivity Median (25%, 75%)
Communication	model 1	0.599 (0.549, 0.649)	0.729 (0.701, 0.757)	0.400 (0.200, 0.500)
	model 2	0.616 (0.567, 0.692)	0.748 (0.720, 0.785)	0.400 (0.200, 0.500)
	model 3	0.629 (0.551, 0.690)	0.766 (0.720, 0.787)	0.400 (0.200, 0.500)
	model 4	0.636 (0.569, 0.701)	0.766 (0.720, 0.794)	0.400 (0.333, 0.600)
	model 5	0.644 (0.581, 0.701)	0.776 (0.738, 0.794)	0.400 (0.200, 0.500)
Gross Motor	model 1	0.577 (0.523, 0.613)	0.721 (0.666, 0.760)	0.250 (0.125, 0.375)
	model 2	0.570 (0.528, 0.611)	0.702 (0.675, 0.740)	0.250 (0.125, 0.375)
	model 3	0.568 (0.532, 0.618)	0.798 (0.760, 0.821)	0.250 (0.125, 0.250)
	model 4	0.561 (0.531, 0.619)	0.779 (0.740, 0.817)	0.250 (0.125, 0.375)
	model 5	0.574 (0.529, 0.622)	0.721 (0.692, 0.750)	0.354 (0.250, 0.375)
Fine Motor	model 1	0.606 (0.549, 0.723)	0.773 (0.732, 0.811)	0.000 (0.000, 0.333)
	model 2	0.620 (0.548, 0.716)	0.709 (0.664, 0.745)	0.000 (0.000, 0.333)
	model 3	0.627 (0.554, 0.713)	0.755 (0.709, 0.782)	0.333 (0.000, 0.500)
	model 4	0.631 (0.550, 0.740)	0.764 (0.734, 0.802)	0.333 (0.000, 0.500)
	model 5	0.670 (0.594, 0.764)	0.800 (0.763, 0.827)	0.333 (0.000, 0.667)
Problem Solving	model 1	0.589 (0.523, 0.689)	0.734 (0.699, 0.789)	0.333 (0.250, 0.500)
	model 2	0.614 (0.541, 0.691)	0.743 (0.706, 0.780)	0.333 (0.000, 0.333)
	model 3	0.631 (0.560, 0.723)	0.780 (0.743, 0.817)	0.333 (0.000, 0.500)
	model 4	0.636 (0.564, 0.720)	0.752 (0.725, 0.799)	0.333 (0.000, 0.333)
	model 5	0.643 (0.550, 0.731)	0.780 (0.743, 0.807)	0.333 (0.000, 0.375)
Personal-Social	model 1	0.571 (0.527, 0.633)	0.733 (0.695, 0.762)	0.250 (0.125, 0.375)
	model 2	0.580 (0.514, 0.623)	0.686 (0.648, 0.726)	0.250 (0.143, 0.375)
	model 3	0.549 (0.505, 0.598)	0.724 (0.686, 0.752)	0.250 (0.125, 0.375)
	model 4	0.569 (0.520, 0.617)	0.686 (0.655, 0.724)	0.250 (0.138, 0.375)
	model 5	0.569 (0.521, 0.624)	0.714 (0.686, 0.743)	0.286 (0.143, 0.429)

**Table 3:** The Grid Search Results of Optimized Integrated Model for Predicting Abnormal Neurodevelopment in Any Area at One Year of Age of Children.

Size ^a^	Decay ^b^	Accuracy Median (25%, 75%)	Sensitivity Median (25%, 75%)	Specificity Median (25%, 75%)	AUC Median (25%, 75%)
4	0.2	0.721 (0.696, 0.739)	0.700 (0.597, 0.797)	0.742 (0.680, 0.748)	0.821 (0.716, 0.833)
6	0.4	0.730 (0.699, 0.746)	0.700 (0.562, 0.738)	0.731 (0.723, 0.757)	0.812 (0.715, 0.832)
2	0.01	0.721 (0.695, 0.734)	0.770 (0.588, 0.837)	0.704 (0.669, 0.753)	0.811 (0.727, 0.833)
3	0.05	0.720 (0.653, 0.747)	0.718 (0.550, 0.825)	0.715 (0.659, 0.741)	0.809 (0.709, 0.823)
6	0.5	0.720 (0.690, 0.763)	0.750 (0.575, 0.750)	0.726 (0.712, 0.749)	0.808 (0.722, 0.829)
2	0.4	0.735 (0.701, 0.765)	0.700 (0.550, 0.738)	0.751 (0.734, 0.761)	0.808 (0.713, 0.829)
4	0.4	0.733 (0.701, 0.767)	0.693 (0.532, 0.750)	0.753 (0.699, 0.770)	0.808 (0.708, 0.830)
8	0.3	0.719 (0.688, 0.749)	0.641 (0.545, 0.788)	0.726 (0.710, 0.748)	0.807 (0.713, 0.821)
7	0.3	0.717 (0.684, 0.740)	0.718 (0.550, 0.788)	0.726 (0.680, 0.747)	0.806 (0.705, 0.825)
4	0.5	0.732 (0.684, 0.756)	0.700 (0.512, 0.738)	0.735 (0.704, 0.772)	0.806 (0.713, 0.828)

^a^ size: This parameter refers to the number of neurons within the hidden layer of the network. Determine the complexity of the model. ^b^ decay: The decay parameter is associated with the regularization process. By adjusting the decay parameters, the model achieves a good balance between underfitting and overfitting.

**Table 4:** SHAP value of the variables in the five predictive models and the number of occurrences of the variables.

Variable Name	Mean(|SHAP Value|)					Num
	Communication	Gross Motor	Fine Motor	Problem Solving	Personal-Social	
Family income	0.095	0.083	-	0.125	0.088	4
Sum of people	0.060	0.099	-	0.070	0.057	4
Chemical exposure	0.059	-	-	0.060	0.111	3
Maternal pre-pregnancy BMI	0.057	0.062	-	-	-	2
Acetaminophen	-	0.073	-	-	-	1
Blue Scutellaria Oral Liquid	-	-	-	0.057	-	1
Calcium Acetate Granules	-	-	-	-	0.086	1
Chuanbai Anti-Itch Wash	-	0.062	-	-	-	1
Currently supplemented folate	-	-	-	0.075	-	1
Ferrous Succinate	-	-	0.080	-	-	1
Hydroxyethyl Starch Sodium Chloride Injection	-	-	0.062	-	-	1
Midazolam injection	-	-	0.097	-	-	1
Physical exposure	-	-	-	-	0.060	1
PSQI	0.104	-	-	-	-	1
Ropivacaine mesylate injection	-	-	0.097	-	-	1
Subthreshold anxiety	-	-	0.068	-	-	1

## Data Availability

The data used in this study are not available publicly due to data protection regulation.

## Supplementary Materials

The following supporting information can be downloaded at: https://www.mdpi.com/article/10.3390/healthcare12070713/s1, Table S1. Predictive performance of different models with full variables under SMOTE oversampling methods in Communication area. Table S2. Predictive performance of different models with full variables under SMOTE oversampling methods in Gross Motor area. Table S3. Predictive performance of different models with full variables under SMOTE oversampling methods in Fine Motor area. Table S4. Predictive performance of different models with full variables under SMOTE oversampling methods in Problem Solving area. Table S5. Predictive performance of different models with full variables under SMOTE oversampling methods in Personal-Social area. Figure S1. Importance of variables in Communication Area. Figure S2. Importance of variables in Gross Motor Area. Figure S3. Importance of variables in Fine Motor Area. Figure S4. Importance of variables in Problem Solving Area. Figure S5. Importance of variables in Personal-Social Area.
