# Supplementary material for: Medication Usage Record-Based Predictive Modeling of Neurodevelopmental Abnormality in Infants under One Year: A Prospective Birth Cohort Study
Source: Healthcare (Basel). 2024 Mar 24;12(7):713. doi: 10.3390/healthcare12070713 (PMC11011488; doi:10.3390/healthcare12070713)
Supplement: Supplementary file 1 [file healthcare-12-00713-s001.zip › healthcare-2855491-supplementary.pdf]

Supplement

Table S1. Predictive performance of different models with different variables under different oversampling methods in Communication area

Oversampling method	Model	Logistic				SVM				RF				ANN			
		Accu	Sens	Spec	AUC	Accu	Sens	Spec	AUC	Accu	Sens	Spec	AUC	Accu	Sens	Spec	AUC
Up-Sampling	model 1	0.618	0.273	0.636	0.613	0.773	0.000	0.813	0.634	0.893	0.091	0.935	0.589	0.764	0.000	0.804	0.630
	model 2	0.644	0.273	0.664	0.596	0.889	0.000	0.935	0.545	0.929	0.182	0.967	0.627	0.800	0.182	0.832	0.517
	model 3	0.676	0.091	0.706	0.617	0.916	0.000	0.963	0.556	0.889	0.000	0.935	0.511	0.836	0.000	0.879	0.523
ROSE	model 1	0.644	0.273	0.664	0.611	0.760	0.000	0.799	0.572	0.778	0.273	0.804	0.587	0.782	0.091	0.818	0.736
	model 2	0.662	0.182	0.687	0.628	0.773	0.000	0.813	0.501	0.813	0.182	0.846	0.579	0.818	0.273	0.846	0.494
	model 3	0.689	0.182	0.715	0.599	0.880	0.091	0.921	0.456	0.898	0.000	0.944	0.769	0.769	0.182	0.799	0.486
SMOTE	model 1	0.702	0.364	0.720	0.516	0.729	0.273	0.752	0.531	0.769	0.182	0.799	0.606	0.702	0.455	0.715	0.602
	model 2	0.684	0.364	0.701	0.562	0.733	0.364	0.752	0.489	0.782	0.273	0.808	0.644	0.716	0.364	0.734	0.636
	model 3	0.738	0.273	0.762	0.556	0.773	0.091	0.808	0.471	0.911	0.000	0.958	0.503	0.747	0.091	0.780	0.548

Table S2. Predictive performance of different models with different variables under different oversampling methods in Gross Motor area

Oversampling method	Model	Logistic				SVM				RF				ANN			
		Accu	Sens	Spec	AUC	Accu	Sens	Spec	AUC	Accu	Sens	Spec	AUC	Accu	Sens	Spec	AUC
Up-Sampling	model 1	0.571	0.500	0.577	0.471	0.768	0.250	0.808	0.599	0.871	0.000	0.938	0.556	0.692	0.250	0.726	0.529
	model 2	0.562	0.312	0.582	0.636	0.768	0.125	0.817	0.586	0.897	0.000	0.966	0.503	0.710	0.312	0.740	0.512
	model 3	0.688	0.250	0.721	0.597	0.897	0.062	0.962	0.620	0.911	0.000	0.981	0.508	0.799	0.062	0.856	0.731
ROSE	model 1	0.576	0.438	0.587	0.587	0.714	0.500	0.731	0.639	0.670	0.562	0.678	0.641	0.643	0.438	0.659	0.537
	model 2	0.607	0.312	0.630	0.571	0.723	0.188	0.764	0.499	0.750	0.125	0.798	0.511	0.701	0.375	0.726	0.520
	model 3	0.674	0.250	0.707	0.608	0.817	0.125	0.870	0.624	0.853	0.062	0.913	0.603	0.759	0.188	0.803	0.535
SMOTE	model 1	0.710	0.125	0.755	0.484	0.781	0.312	0.817	0.578	0.812	0.125	0.865	0.589	0.674	0.375	0.697	0.480
	model 2	0.714	0.312	0.745	0.580	0.719	0.125	0.764	0.542	0.808	0.250	0.851	0.646	0.616	0.312	0.639	0.523
	model 3	0.723	0.250	0.760	0.537	0.714	0.062	0.764	0.535	0.772	0.188	0.817	0.593	0.719	0.062	0.769	0.435

Table S3. Predictive performance of different models with different variables under different oversampling methods in Fine Motor area

Oversampling method	Model	Logistic				SVM				RF				ANN			
		Accu	Sens	Spec	AUC	Accu	Sens	Spec	AUC	Accu	Sens	Spec	AUC	Accu	Sens	Spec	AUC
Up-Sampling	model 1	0.705	0.400	0.712	0.532	0.942	0.000	0.963	0.571	0.955	0.000	0.977	0.539	0.924	0.000	0.945	0.568
	model 2	0.705	0.400	0.712	0.506	0.973	0.000	0.995	0.712	0.978	0.000	1.000	0.536	0.924	0.000	0.945	0.654
	model 3	0.781	0.200	0.795	0.494	0.969	0.000	0.991	0.563	0.955	0.000	0.977	0.555	0.938	0.000	0.959	0.766
ROSE	model 1	0.737	0.400	0.744	0.528	0.884	0.000	0.904	0.622	0.906	0.000	0.927	0.463	0.871	0.000	0.890	0.621
	model 2	0.728	0.400	0.735	0.549	0.906	0.000	0.927	0.509	0.929	0.000	0.950	0.552	0.857	0.200	0.872	0.564
	model 3	0.857	0.200	0.872	0.552	0.960	0.000	0.982	0.500	0.964	0.000	0.986	0.752	0.893	0.000	0.913	0.577
SMOTE	model 1	0.692	0.600	0.694	0.653	0.826	0.400	0.836	0.727	0.754	0.600	0.758	0.649	0.612	0.600	0.612	0.532
	model 2	0.683	0.600	0.685	0.642	0.705	0.400	0.712	0.601	0.723	0.400	0.731	0.553	0.647	0.800	0.644	0.657
	model 3	0.562	0.600	0.562	0.600	0.746	0.400	0.753	0.668	0.759	0.600	0.763	0.646	0.679	0.600	0.680	0.652

Table S4. Predictive performance of different models with different variables under different oversampling methods in Problem Solving area

Oversampling method	Model	Logistic				SVM				RF				ANN			
		Accu	Sens	Spec	AUC	Accu	Sens	Spec	AUC	Accu	Sens	Spec	AUC	Accu	Sens	Spec	AUC
Up-Sampling	model 1	0.562	0.500	0.564	0.560	0.862	0.167	0.881	0.649	0.955	0.000	0.982	0.751	0.848	0.333	0.862	0.694
	model 2	0.634	0.167	0.647	0.595	0.920	0.167	0.940	0.604	0.960	0.000	0.986	0.702	0.902	0.167	0.922	0.489
	model 3	0.741	0.000	0.761	0.659	0.964	0.000	0.991	0.556	0.951	0.000	0.977	0.536	0.875	0.000	0.899	0.437
ROSE	model 1	0.656	0.000	0.674	0.590	0.790	0.333	0.803	0.667	0.848	0.667	0.853	0.832	0.790	0.333	0.803	0.765
	model 2	0.674	0.167	0.688	0.530	0.866	0.333	0.881	0.726	0.893	0.167	0.913	0.740	0.853	0.000	0.876	0.507
	model 3	0.777	0.333	0.789	0.496	0.929	0.000	0.954	0.511	0.969	0.000	0.995	0.570	0.857	0.000	0.881	0.559
SMOTE	model 1	0.732	0.167	0.748	0.507	0.799	0.167	0.817	0.568	0.839	0.167	0.858	0.563	0.746	0.167	0.761	0.555
	model 2	0.826	0.167	0.844	0.641	0.799	0.167	0.817	0.743	0.906	0.167	0.927	0.642	0.737	0.167	0.752	0.626
	model 3	0.790	0.000	0.812	0.518	0.844	0.167	0.862	0.612	0.911	0.000	0.936	0.625	0.759	0.167	0.775	0.657

Table S5. Predictive performance of different models with different variables under different oversampling methods in Personal-Social area

Oversampling method	Model	Logistic				SVM				RF				ANN			
		Accu	Sens	Spec	AUC	Accu	Sens	Spec	AUC	Accu	Sens	Spec	AUC	Accu	Sens	Spec	AUC
Up-Sampling	model 1	0.513	0.600	0.507	0.547	0.714	0.333	0.742	0.570	0.866	0.000	0.928	0.557	0.728	0.267	0.761	0.505
	model 2	0.549	0.533	0.550	0.488	0.772	0.000	0.828	0.496	0.902	0.000	0.967	0.552	0.710	0.267	0.742	0.469
	model 3	0.598	0.267	0.622	0.498	0.835	0.200	0.880	0.555	0.902	0.000	0.967	0.482	0.848	0.333	0.885	0.641
ROSE	model 1	0.500	0.533	0.498	0.543	0.558	0.467	0.565	0.487	0.670	0.267	0.699	0.514	0.536	0.267	0.555	0.556
	model 2	0.549	0.467	0.555	0.499	0.679	0.400	0.699	0.576	0.714	0.467	0.732	0.575	0.719	0.467	0.737	0.626
	model 3	0.589	0.467	0.598	0.495	0.772	0.133	0.818	0.487	0.835	0.133	0.885	0.446	0.705	0.333	0.732	0.546
SMOTE	model 1	0.705	0.400	0.727	0.504	0.750	0.200	0.789	0.520	0.763	0.067	0.813	0.650	0.612	0.333	0.632	0.523
	model 2	0.679	0.333	0.703	0.511	0.728	0.267	0.761	0.540	0.821	0.067	0.876	0.577	0.652	0.333	0.675	0.548
	model 3	0.670	0.267	0.699	0.555	0.763	0.200	0.804	0.548	0.884	0.067	0.943	0.559	0.723	0.467	0.742	0.677

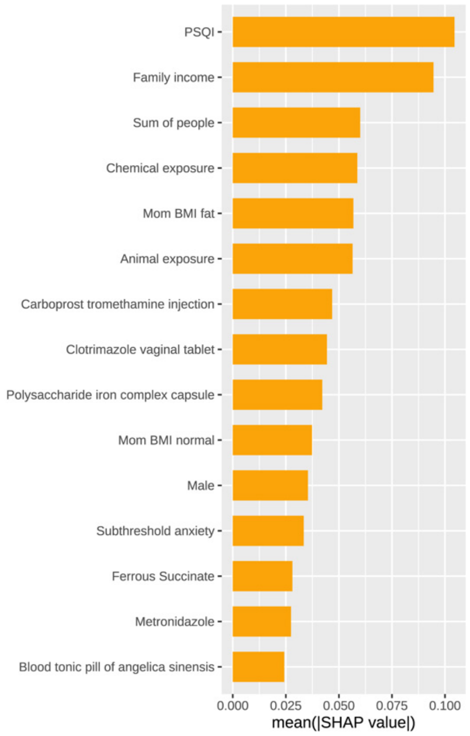

Figure S1. Importance of variables in Communication Area

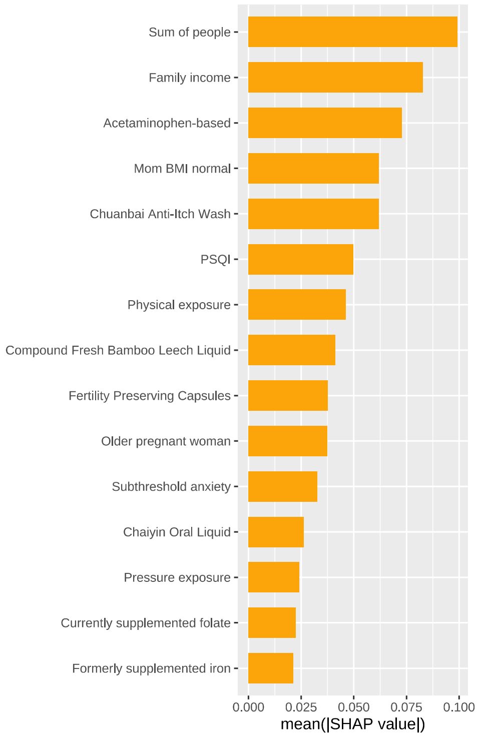

Figure S2. Importance of variables in Gross Motor Area

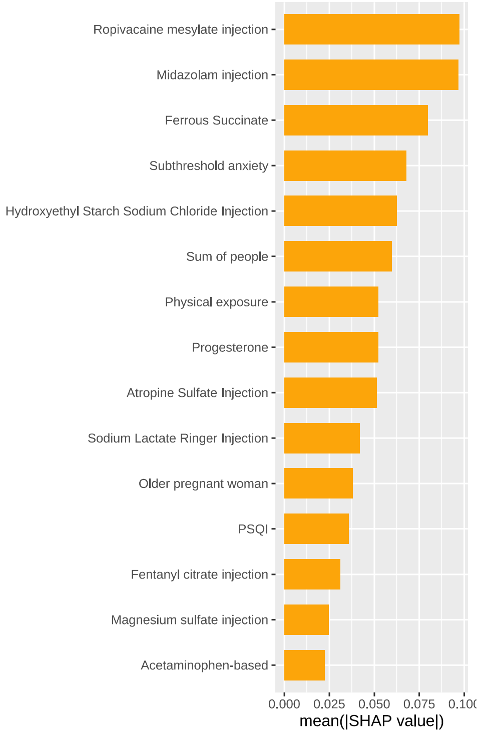

Figure S3. Importance of variables in Fine Motor Area

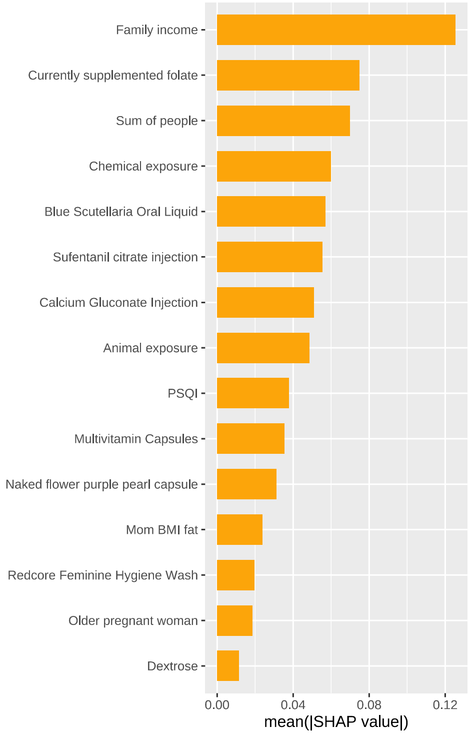

Figure S4. Importance of variables in Problem Solving Area

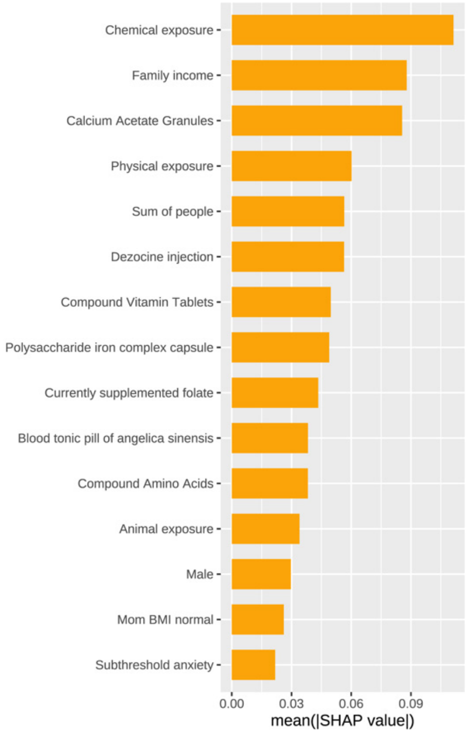

Figure S5. Importance of variables in Personal-Social Area
